# How does rerupture affect sleep and quality of life in patients undergoing arthroscopic rotator cuff repair?

**DOI:** 10.3906/sag-2012-169

**Published:** 2021-02-26

**Authors:** İzzet BİNGÖL, Vedat BİÇİCİ

**Affiliations:** 1 Department of Orthopaedics and Traumatology, 29 Mayıs Goverment Hospital, Ankara Turkey; 2 Department of Orthopaedics and Traumatology, Ankara City Hospital, Ankara Turkey

**Keywords:** Rotator cuff tear, sleep and quality of life, rotator cuff rerupture, Pittsburgh sleep quality index

## Abstract

**Background/aim:**

Sleep disturbance and related improvement in quality of life as a result of arthroscopic repair in rotator cuff tear (RCT) patients can be considered as an important parameter. The aim of our study is to evaluate the rotator cuff by ultrasonography (USG) in the first postoperative year and to examine whether there is a difference between sleep disturbance and quality of life between cases of rupture and healthy patients. In addition, we aim to compare the preoperative and at least the first postoperative year’s sleep disturbances and quality of life among patients who underwent arthroscopic RCT repair and to examine the effects of factors affecting this situation.

**Materials and methods:**

We retrospectively reviewed the records of patients who were operated on for RCT. In this process, 257 patients were examined and 76 patients who met the inclusion criteria were included in the study. The Pittsburgh sleep quality index (PSQI), Constant–Murley shoulder score (CSS), and Oxford shoulder score (OSS) were used to evaluate the results preoperatively and at the last control visit of each patient. In the USG performed in the postoperative first year, the rotor cuff was evaluated in terms of rerupture.

**Results:**

It was observed that 14 (18.4%) patients’ rotor cuffs were reruptured and those of 62 (81.6%) patients were intact. The preoperative PSQI, CSS, and OSS values of the patients were calculated as an average of 10.79 ± 3.58, 35.61 ± 8.88, and 17.61 ± 4.20 and the mean postoperative values were calculated as 5.45 ± 1.68, 81.55 ± 5.27, and 38.05 ± 3.06, respectively. The postoperative PSQI value was statistically significantly lower in patients with tears of <1 cm (PSQI: 4.29 ± 0.73) than in those with tears of 1–3 cm (PSQI: 5.50 ± 2.17) and 3–5 cm (PSQI: 5.88 ± 1.25) (P < 0.001). The mean CSS and OSS values were significantly higher in postoperative measurements for all tear types. According to the size of the tear, postoperative CSS and OSS values were statistically significantly lower in patients with tears of 3–5 cm (CSS: 78.59 ± 4.50 and OSS: 36.18 ± 2.47) than those with <1 cm (CSS: 85.43 ± 2.14 and OSS: 40.57 ± 1.55) and 1–3 cm (CSS: 83.21 ± 5.35 and OSS: 39.07 ± 2.94) tears (P < 0.001).

**Conclusion:**

In the USG performed in the postoperative first year, it was determined that the patients with healthy rotator cuffs recovered better than those with rerupture.

## 1. Introduction

Rotator cuff tear (RCT) is the second most common musculoskeletal pathology and one of the most common shoulder ailments for which patients need treatment [1,2]. RCT causes pain, weakness, stiffness, and limitation of movement in the shoulder [3]. Sleep disturbance, especially caused by night pain, is an important source of complaints in patients, but the exact degree of pain that prevents sleep is unknown [4,5]. This problem may cause negative emotional, behavioral, and cognitive effects in the patient and increase the prevalence of anxiety and depression [6,7]. According to the American Academy of Sleep Medicine and the Sleep Research Society, regularly sleeping fewer than 7 h per night can cause health problems such as obesity, diabetes mellitus (DM), hypertension (HT), heart disease, stroke and depression [7,8]. These problems alone are sufficient to create the need for treatment in a patient [9]. Adequate sleep is important for the recovery and satisfaction of the patient, and sleep disturbance and related improvement in quality of life as a result of arthroscopic repair in RCT patients can be considered as an important parameter.

Arthroscopic RCT repair can generally improve shoulder pain, joint functions, psychological status, physical function, and general health status, but it may not provide sleep recovery in every patient due to patient-specific pathologies such as psychiatric disorders [9–11]. It has been shown that sleep disorders occur in between 15% and 35% of the general population [9,12]. There may be different factors such as functional limitations, challenging positions, and shoulder stiffness that play important roles in reducing sleep quality [9]. In addition, it was concluded that preoperative and postoperative use of narcotic pain medications increased wakefulness and inhibited REM sleep, resulting in sleep disturbance [9,13].

There is no consensus on improving sleep disturbance and quality of life after arthroscopic RCT repair. To date, it has been shown that there are relationships between sleep quality and factors such as female gender, HT, DM, body mass index (BMI), depression, low back pain, corticosteroid injections, and osteoarthritis in patients with RCT, while no statistically significant relationship was found between RCT features and sleep quality [7,12,14]. When the literature is reviewed, there is no study conducted to investigate the effect of rotator cuff (RC) rerupture on sleep and quality of life in the postoperative period [12].

The aim of our study is to evaluate RCs by ultrasonography (USG) in the first postoperative year and to examine whether there is a difference between sleep disturbance and quality of life between cases of rupture and healthy patients. In addition, we aim to compare the preoperative and at least the first postoperative year’s sleep disturbances and quality of life among patients who underwent arthroscopic RCT repair and to examine the effects of factors affecting this situation. Our hypothesis in this study was as follows: With the USG performed in the postoperative first year, the improvement in sleep and quality of life will be significantly higher in patients with healthy RCs compared to those with ruptures.

## 2. Materials and methods

### 2.1. Study design and participants

We retrospectively reviewed the records of patients who were operated on for RCT by the same surgeon in a single center between 2015 and 2019. In this process, 257 patients treated surgically for RCT were examined. The study’s inclusion criteria were as follows: Patients over 18 years of age who underwent arthroscopic repair due to RCT; atraumatic rerupture without implant failure and without revision surgery according to the Cofield classification [15]; and <1 cm (small), 1–3 cm (medium), or 3–5 cm (large) full-thickness RCT in patients who underwent arthroscopic repair with at least 1 year of follow-up. Exclusion criteria were as follows: Patients who had undergone previous shoulder surgery; massive (>5 cm) RCT; simultaneous adhesive capsulitis; advanced glenohumeral arthritis; history of psychiatric illness; treatment of history for a sleep disorder; use of narcotic analgesics or tenodesis for biceps pathology; and the unwillingness to be included in the study. As a result, 76 patients who met the criteria were included in the study.

The study protocol was approved by the Ethics Committee of Ankara City Hospital (reference number: E1-20-1070).

In this study, the Pittsburgh sleep quality index (PSQI) [16], Constant–Murley shoulder score (CSS) [17], and Oxford shoulder score (OSS) [18] were used to evaluate the results preoperatively and at the last control visit of each patient. Demographic data of the patients such as BMI [19] and nicotine and alcohol use were collected along with age, sex, affected side, chronic diseases [DM, HT, hyperlipidemia (HL)], height, and weight. In the USG performed because it is easier and less costly in the postoperative first year, the RC was evaluated in terms of rerupture.

### 2.2. Perioperative management

All patients were operated on in the sunbed position under hypotensive (systolic blood pressure: 80–100 mmHg) general anesthesia, keeping the joint pump pressure around 50 mmHg. Postoperative intravenous analgesic was administered to patients in whom an interscalene block could not be performed. According to the Cofield classification, the definition of the tear, repair technique, materials used, additional interventions such as subacromial decompression, distal clavicle excision, biceps intervention, and intraoperative complications were recorded after surgery. For tear sizes, AP lengths were measured by arthroscopic imaging using a calibrated probe. Considering the tension of the RC, a single row or transosseous-equivalent double row was repaired.

### 2.3. Postoperative management

Oral analgesics and nonsteroidal antiinflammatory drugs (NSAIDs) were prescribed at the time of discharge. A similar rehabilitation protocol was applied for all patients postoperatively and a shoulder/arm sling was used for immobilization for 4 weeks. Pendular exercise was started on the postoperative first day. On the 15th day, the sutures were removed and four-way isometric exercises and passive exercises of up to 90° were started. In addition to isometric exercises, active exercises of 90° and passive exercises of 90–120° were added in the first month’s control visits. Active full-range motion exercises were started after the eighth week. From the third month, strengthening exercises against resistance were started. After the fourth month, it was possible to return to daily activities without any restrictions.

### 2.4. Study measurements

Patient outcome scores were collected and scored by one research coordinator and were recorded preoperatively and postoperatively.

#### 2.4.1. PSQI

The PSQI is designed to measure sleep quality [16]. It has been generally used in evaluating sleep quality in clinical practice. Patients complete a 19-question survey, from which 7 component scores are obtained: subjective sleep quality, sleep latency, sleep duration, habitual sleep efficiency, sleep disorder, sleep medication use, and daytime functional disorder. This questionnaire yields scores from 0 to 21 points. A higher score indicates worse sleep dysfunction and a total score of >5 is indicative of poor sleep quality.

#### 2.4.2. CSS

The CSS is a comprehensive and comparable assessment of shoulder function [17]. This patient and clinician completed survey contains four subscales: pain (15 points), activities of daily living (20 points), strength (25 points), and range of motion including forward elevation, external rotation, abduction, and internal rotation of the shoulder (40 points). A higher score represents higher quality of function.

#### 2.4.3. OSS

The OSS aims to provide a self-assessment of pain and function of the shoulder and is commonly used in shoulder operations other than stabilization [18]. It comprises 12 items: 4 about pain (2 for pain, 2 for interference with pain) and 8 about daily functions. Each item in the original scale is scored according to a 5-point Likert-type scale, where 1 = no pain/easy to do, 2 = mild pain/little difficulty, 3 = moderate pain/moderate difficulty, 4 = severe pain/extreme difficulty, and 5 = unbearable/impossible to do. In the revision study and the online form, the item scoring is from 0 (worst) to 4 (best).

### 2.5. Statistical analysis

Data analyses were performed using SPSS 22.0 for Windows (IBM Corp., Armonk, NY, USA). Whether the distribution of continuous variables was normal or not was determined by the Kolmogorov–Smirnov test. Levene’s test was used for the evaluation of homogeneity of variances. Unless otherwise specified, continuous data were described as mean ± SD for normal distributions and as mean ± SD and median (interquartile range) for skewed distributions. Categorical data were described as number of cases (%). In the statistical analysis, differences between nonnormally distributed variables in two independent groups were compared with the Mann–Whitney U test while the differences between nonnormally distributed variables among more than two independent groups were analyzed by the Kruskal–Wallis test. When the P-values from Kruskal–Wallis test statistics were statistically significant, posthoc Conover–Iman tests/multiple comparison tests were used to determine which group differed from the others. Statistically significant differences for nonnormally distributed variables between two dependent groups were compared with the Wilcoxon test. Categorical variables were compared using Pearson’s chi-square test or Fisher’s exact test. Degrees of relation between variables were evaluated with Spearman’s correlation analysis. Multivariate linear regression (backward method) was first used with risk factors thought to be related to postoperative PSQI, OSS, and CSS values. R2 values were analyzed to determine how much independent variables explained dependent variables. The model adaptation of estimates was also analyzed with the ANOVA test model adaptation test. ROC curve analysis was used to determine the cutoff points. Values of P < 0.05 were accepted as significant in all statistical analysis.

## 3. Results

The average age of our patients was 60.39 ± 9.04 years, with 28 men and 48 women. The demographic characteristics of the patients are presented in the Table 1. It was observed that 14 (18.4%) patients’ RCs was reruptured and those of 62 (81.6%) patients were intact. Those with rerupture had statistically significantly higher postoperative PSQI scores and lower CSS and OSS scores compared to those without rerupture (P < 0.001) (Table 2).

**Table 1:** Demographic information of patients (n: 76).

Age, years, x̄ ± SD median (IQR)		60.39 ± 9.04 61.5 (11)
Sex, male/female		28/48
Body mass index, x̄ ± SD median (IQR)		31.44 ± 4.67 31.4 (5.59)
Passing time, months, x̄ ± SD median (IQR)		35.16 ± 16.91 30.5 (33)
Operated side, right/left, n		40/36
Trauma, n (%)		24 (31.6%)
Chronic disease (DM, HT, HL), n		22, 34, 8
Smoker, n (%)		12 (15.9%)
Alcohol, n (%)		2 (2.6%)
Narcotics, n (%)		-
Tear size, cm	<1 cm	14 (18.4%)
	1–3 cm	28 (36.8%)
	1–5 cm	34 (44.7%)
Surgery technique	DR	26 (34.2%)
	SR	50 (65.8%)
Postoperative USG	Rerupture	14 (18.4%)
	Healthy	62 (81.6%)

Continuous variables are expressed as mean ± standard deviation (SD) and/or median (IQR). Categorical variables are expressed as either frequency or percentage.

**Table 2:** Postoperative USG and patients’ scores.

	Rerupture (n:14)	Healthy (n: 62)	
Postop PSQI	7.29 ± 2.337 (3)	5.03±1.165 (2)	<0.01
Postop CSS	73.43 ± 4.9374 (10)	83.39±3.2484 (4)	<0.001
Postop OSS	33.71 ± 2.6434 (5)	39.03 ± 2.1839 (2)	<0.001

Continuous variables are expressed as mean ± standard deviation (SD) and median (interquartile range). Independent continuous variables were compared with the Mann–Whitney U test. Statistically significant P-values are in bold.

The postoperative PSQI value among all patients was thus statistically significantly lower than the preoperative value (P < 0.001). The postoperative CSS value among all patients was statistically significantly increased compared to the preoperative value (P < 0.001). The postoperative OSS value increased statistically significantly among all patients compared to the preoperative value (P < 0.001) (Table 3).

**Table 3:** Scores of patients.

	Preop	Postop	P
PSQI	10.79 ± 3.5812 (7)	5.45 ± 1.685 (1)	<0.001
CSS	35.61 ± 8.8834 (8)	81.55 ± 5.2782 (4)	<0.001
OSS	17.61 ± 4.2017 (4)	38.05 ± 3.0638 (3)	<0.001

Continuous variables are expressed as mean ± standard deviation (SD) and median (interquartile range). Dependent continuous variables were compared with the Wilcoxon test. Statistically significant P-values are in bold.

According to the RCT Cofield classification, 25 patients had ruptures of <1 cm (small), 23 patients of 1–3 cm (medium), and 13 patients of 3–5 cm (large). Sleep disturbances had improved significantly in the postoperative measurements for all tear types. According to the size of the tears, the postoperative PSQI value was statistically significantly lower in patients with tears of <1 cm than those with tears of 1–3 cm and 3–5 cm (P < 0.001). The mean CSS and OSS values were significantly higher in the postoperative measurements of all tear types. According to the size of the tear, postoperative CSS and OSS values were statistically significantly lower in patients with tears of 3–5 cm than those with tears of <1 cm and 1–3 cm (P < 0.001) (Table 4).

**Table 4:** Tear size and patients’ scores.

	<1 cm (n: 14)	1–3 cm (n: 28)	3–5 cm (n: 34)	
Postop PSQI	4.29 ± 0.734 (1)	5.50 ± 2.175 (2)	5.88 ± 1.256 (2)	<0.001a,b
Postop CSS	85.43 ± 2.1486 (4)	83.21 ± 5.3584 (4)	78.59 ± 4.5080 (6.5)	<0.001b,c
Postop OSS	40.57 ± 1.5541 (3)	39.07 ± 2.9439.5 (2)	36.18 ± 2.4737 (2.5)	<0.001b,c

Continuous variables are expressed as mean ± standard deviation (SD) and median (interquartile range). Independent continuous variables were compared with the Kruskal–Wallis test. The Conover–Inman test was performed for binary comparisons among the groups and the P-value was set at 0.05. Significant differences were found between: a: <1 cm vs. 1–3 cm, b: <1 cm vs. 3–5 cm, c: 1–3 cm vs. 3–5 cm. Statistically significant P-values are in bold.

It has been observed that alcohol use affects postoperative PSQI results. Specifically, alcohol use increased the postoperative PSQI score in this study (P = 0.015). None of the patients used narcotics. The variables of age, sex, follow-up time, surgery side, BMI, trauma history, DM, HT, HL, smoking, and surgical technique did not independently affect sleep disorder (i.e. were statistically insignificant). No complication that might require treatment developed in any of our patients during the postoperative period.

## 4. Discussion

When the literature is reviewed, there is no study conducted to investigate the effect of rerupture and robustness of the RC on the quality of sleep and life in the postoperative period of patients who have undergone arthroscopic repair due to RCT [12]. When patients with rupture and those without were compared in our study, it was found that those with rupture had lower sleep quality.

Although many studies have been reported on the relations between shoulder pathologies such as subacromial impingement, glenohumeral arthrosis, and adhesive capsulitis and sleep disturbance, only a few studies have been reported on the effects of RCT on sleep disturbance [1,11]. Cho et al. reported sleep disturbance (PSQI: 8.1) caused by chronic pain in RCT patients [20]. Mulligan et al. reported that 93% of patients with RCT had night pain and 71% had sleep disturbance (PSQI: >5), and the cause of the sleep disturbance was related to night pain [5]. Gumina et al. claimed that RCT is only one of the causes of sleep disturbance in middle and old ages. In addition, they reported that night pain was the main cause of sleep disorders in RCT patients [21]. Khazzam et al. stated that 94% of patients with RCT complained of night pain (PSQI: 9.48), and 89% of patients with RC tendinitis (impingement) had night pain (PSQI: 8.66). They stated that shoulder pain symptoms may not be clearly related to the severity of RC disorders [7]. Our present findings for patients with RCT are compatible with those presented in the literature, with an average PSQI value of 10.79 ± 3.58. This result shows a correlation between sleep disturbance and RCT.

Recovery from sleep disturbance after arthroscopic RCT repair was first described in the literature by Austin et al. [9], who stated that sleep disturbance (PSQI: >5) decreased from 89% preoperatively to 38% in the sixth postoperative month and that recovery continued for 6 months. Cho et al. reported that sleep disturbance improved in the 12-month follow-up of 47 patients who underwent RCT repair, but for 42% of the patients, problems still continued (PSQI: >5) [20]. Serbest et al. found a dramatic improvement in sleep disturbance in a 6-month follow-up period (PSQI: 15 to 6) for 31 patients who underwent RCT repair. Although they found a great improvement in sleep disturbance, 58% of patients still complained of sleep disturbance (PSQI: >5) 6 months after surgery [22]. Hornef et al. reported that 89% of RCT patients with preoperative sleep disorders (PSQI: 11.7) recovered postoperatively, while41% (PSQI: 5.5) recovere after at least 24 months of follow-up [23]. In our study, it was observed that the mean PSQI score decreased from 10.79 ± 3.58 to 5.45 ± 1.68. The mean follow-up time was 31.44 ± 4.67 months, which was longer than in previous studies. In this case, it was observed that there was a great improvement in sleep disturbance after at least 1 year of follow-up after arthroscopic RCT repair. We can thus say that sleep disturbance decreases after arthroscopic RCT repair.

According to Austin et al., RCT dimensions did not correlate with sleep disorders [9]. Supporting this study, there are many studies showing that tear size does not affect sleep disorder [7,20,22,23]. Gumina et al. reported that patients with small tears had lower sleep quality than those with larger tears. However, in this study, sleep disturbance was reported only in the preoperative period, and sleep improvement was not reported in the postoperative period [21]. According to the study of Cicekli, large tears caused lower sleep quality than other tears [24]. In our study, tears of 3–5 cm (large) caused lower sleep quality than other tears.

Austin et al. demonstrated correlations among the simple shoulder test (SST), visual analog scale for pain (VAS), and PSQI. An increase in SST was accompanied by decreases in VAS and PSQI [9]. Horneff et al. reported that the correlation between SST, VAS, and PSQI scores continued for up to 24 months [23]. Serbest et al. found a correlation between shoulder scores and sleep disturbance [22]. In our study, the improvements in shoulder scores and sleep quality were associated. Good results in PSQI scores together with good results in CSS and OSS scores have been demonstrated after at least 1 year of follow-up.

Khazzam et al. showed that factors causing sleep disturbance in cases of RCT are female gender, depression, low back pain, DM, cervical involvement, and high BMI, but they did not examine the factors affecting the results of RCT repair [7]. Austin et al. stated that the only independent demographic or surgical factor affecting postoperative sleep was the use of narcotic pain medication [9]. Serbest et al. reported that there was no correlation between demographic variables and chronic disease and sleep disturbance in patients who underwent RCT repair [22]. Additionally, less improvement in sleep quality after RCT repair was reported in patients with concurrent DM and HT [24]. In our study, demographic data of the patients such as age, sex, follow-up time, surgery side, BMI, trauma history, DM, HT, HL, smoking, and surgical technique were analyzed, and it was found that there was no effect on sleep disorders after RCT repair.

The most important feature of our study is the evaluation of RCs with USG in the first postoperative year among patients who underwent arthroscopic repair due to RCT. We consider the exclusion of patients who underwent previous shoulder surgery or had massive (>5 cm) RCT, simultaneous adhesive capsulitis, advanced glenohumeral arthritis, psychiatric disease history, history of treatment for sleep disorder, use of narcotic analgesics, and tenodesis for biceps pathology as an advantage of this study. The limitations of this study are that it was retrospective and there was not a control group or isolated RCT patient group. It is necessary to study patients with the same types of tears belonging to the same sex and similar age groups together with a control group comprising individuals who do not have additional shoulder pathologies or chronic diseases with RCT.

In this study, in the USG performed in the first postoperative year, it was determined that the patients with healthy rotator cuffs recovered better than those with ruptures. According to this, we can say that in the follow-up of patients who underwent arthroscopic repair due to RCT, patients with intact RC have more significant improvement in sleep disturbance and quality of life compared to patients with ruptures. Due to the limitations of this study, it is difficult to say that the sleep disorders of patients who underwent RCT arthroscopic repair recovered specifically due to that repair. However, we can say that sleep disturbance and quality of life improved as a result of the arthroscopic repair performed for RCT and its accompanying pathologies and the rehabilitation protocol applied during the follow-up process.
